# Evolution of total wrist arthroplasty

**DOI:** 10.1186/1753-6561-9-S3-A87

**Published:** 2015-05-19

**Authors:** Philippe Liverneaux

**Affiliations:** 1 Department of Hand Surgery, Strasbourg University Hospitals, F-67493, Illkirch, France

## 

Since the first wrist implant in 1890 by Glück in the form of an ivory prosthesis, the evolution of the prosthetic wrist has been sawtoothing. Abandoned in the late nineteenth century due to infectious complications, they reappeared after the Second World War. Several concepts have rubbed shoulders for decades. It flourished simple and lightweight silicone implants Swanson and complex and heavy metal prostheses Meuli kind. Silicone implants gave spectacular results in the early years but were abandoned due to siliconite and / or implant fracture. The metallic prostheses can be categorised into multiple implants or "ball and socket", cemented or not, inverted or not, with or without polyethylene or retentive unconstrained. Several generations have been developed to reduce complications. However, regardless of the type of prosthesis, survival at 5 years did not exceed 50% mainly due to the loosening of the carpal component.

This is what has led to the arrival of new total prostheses in the 2000s: the Remotion SBI ® and ™ Uni2 ® Integra ™. These new prostheses preserve the carpal bone stock and seem to have a better survival rate at 5 years than the previous generation.

A new concept that is emerging in recent years, is the concept of unicompartmental prosthesis. Assuming that the loosening of the carpal component is the main problem of total prostheses, deletion avoids this complication. The mechanical stresses are lower than the hip, so therefore the theoretical risk in the radial carpal prosthesis is low. It is still too early to comment on the results. Nevertheless, the evolution of prosthetic wrist is not complete, as evidenced by the most recent implants.

